# [Retracted] Zearalenone regulates endometrial stromal cell apoptosis and migration via the promotion of mitochondrial fission by activation of the JNK/Drp1 pathway

**DOI:** 10.3892/mmr.2024.13363

**Published:** 2024-10-15

**Authors:** Huixiang Wang, Xiaoli Zhao, Chengxiang Ni, Yuyang Dai, Yan Guo

Mol Med Rep 17: 7797–7806, 2018; DOI: 10.3892/mmr.2018.8823

Following the publication of this article, a concerned reader drew to the Editor's attention that the experimental design of the western blot assay experiments portrayed in Fig. 5A on p. 7804, and the overall organization of this figure, may have been flawed, as mitochondrial and cytosolic proteins were featured in the figure with only one set of supporting control western blot data; in this scenario, the proteins would necessarily have needed to have been obtained from two separate cell samples in different experiments, and blotted on to separate gels. Moreover, there was also a concern that certain of the gels featured possible breaks in their continuity/splicing events, such that the protein bands in the figure were not shown consecutively, as they would have appeared, in the affected slices.

After having conducted an internal investigation, the Editor of *Molecular Medicine Reports* agrees with the reader that there were anomalies associated with the presentation of Fig. 5. Therefore, on the grounds of a lack of confidence in the presented data, the Editor has decided that the article should be retracted from the publication. The authors were asked for an explanation to account for these concerns, but the Editorial Office did not receive a reply. The Editor apologizes to the readership for any inconvenience caused, and we also thank the reader for bringing this matter to our attention.

